# Neural tube defects in the Free State province from 2012 to 2016. Is there an increase?

**DOI:** 10.4102/sajhivmed.v21i1.1134

**Published:** 2020-09-25

**Authors:** Nické Theron, Gina Joubert, Bertram D. Henderson

**Affiliations:** 1 Department of Paediatrics and Child Health, Faculty of Health Sciences, University of the Free State, Bloemfontein, South Africa; 2 Department of Biostatistics, Faculty of Health Sciences, University of the Free State, Bloemfontein, South Africa; 3 Division of Clinical Genetics, Faculty of Health Sciences, University of the Free State, Bloemfontein, South Africa

**Keywords:** neural tube defects, birth defects, data collection, Free State province, South Africa, antiretroviral treatment

## Abstract

**Background:**

Neural tube defects (NTDs) are anomalies of the central nervous system caused by the defective closure of the neural tube during early embryogenesis. A significant decline in the incidence of NTDs after folic acid fortification of food in South Africa was previously shown. Recently, clinical geneticists have voiced concerns that there is a possible resurgence in the number of NTDs.

**Objectives:**

The aim of this study was to determine the incidence of NTDs at a South African Hospital from 2012 to 2016.

**Methods:**

This is a retrospective cross-sectional study where all babies with NTDs born in, or referred to Universitas Hospital were included as study participants. Information was collected for both the mother and the baby from hospital records and data forms.

**Results:**

Seventy-seven cases of NTDs were captured from 2012 to 2016. The incidence of NTDs was 0.34/1000 births in the Free State province, and 1.21/1000 births if only the data for babies born in Universitas Hospital and Pelonomi Hospital were used. Further analysis showed a male: female ratio of 1:1. Open spina bifida was the most common defect at 71.4%.

**Conclusion:**

The incidence of NTDs in the Free State province was low compared to other South African and international studies. The incidence for the metropolitan hospitals is comparable to that of previous studies. This discrepancy is a marker of poor data recording and will impact healthcare planning. A statistically significant increase in NTDs could not be proven.

## Introduction

Neural tube defects (NTDs) are severe anomalies of the central nervous system caused by the defective closure of the neural tube during early embryogenesis.^1^ An estimated minimum of 300 000 neonates are affected worldwide each year.^2^ According to UNICEF and the South African Burden of Disease study in 2000,^3^ numerous different congenital defects are ranked in the first 20 specific-causes of under-five childhood mortality in South Africa (SA). The specific disorders were congenital heart disease (8th), NTDs (12th), chromosomal defects (18th) and congenital disorders of the gastrointestinal tract (19th).

Despite many studies and growth in the knowledge of neural tube embryology, this multifactorial disorder and the complex genetic and environmental factors interactions that cause or prevent it remain poorly understood.^4^

Neural tube defects are classified as open or closed (membrane-covered) lesions. The type of lesion determines the clinical impact. Open lesions affecting the brain such as anencephaly (absence of major part of the brain, skull and scalp) and craniorachischisis (anencephaly and NTDs that extend to the neck) are lethal antenatally or shortly after birth. The morbidity of encephalocoeles (protrusion of either brain tissue, its covering membranes or both through a defect in the skull) depends on the extent, position and contents of the protrusion. Spina bifida is an NTD that is restricted to the caudal portion of the neural tube. Its morbidity depends on the extent and level of the lesion and the degree of neurological impairment below that level.^5^ Spina bifida can be divided into three common types. Spina bifida occulta results from the failure of vertebral fusion and has no opening on the back (also called a closed NTD). There might be a clue to the defect in the form of a tuft of hair or a dimple in the overlying skin. This is the mildest form and usually does not cause any disability. A protruding sac filled with fluid that does not involve the spinal cord is called a meningocoele. Myelomeningocoele is the most common and severest form. This involves incomplete vertebral and neural tube closure and thus exposure of the spinal cord and meninges which may be open or closed.^6^ This necessitates early surgical treatment of the lesion and the associated complications (such as hydrocephalus) and requires long-term rehabilitation and follow-up.^7^

Numerous risk factors have been identified for NTDs, including both genetic and environmental factors. Maternal vitamin B12 deficiency has been shown to increase the risk of NTDs.^2^ Maternal exposure to teratogens, such as methotrexate, valproic acid, other anticonvulsants and aminopterin, as well as hyperthermia early in pregnancy, low socioeconomic status, maternal obesity, pre-gestational diabetes and genetic predisposition are factors that increase the risk.^4^ Neural tube defects can also occur as part of genetic syndromes as one of the multiple congenital malformations.^1,2^

Since the seminal work of Czeizel and Dudás,^8^ folic acid (vitamin B9) has become the accepted norm for primary prevention of NTDs.^4,7^ Sustainable progress has been made in the primary prevention of NTDs resulting from folic acid supplementation/food fortification. One of the first studies to support this was done in China from 1993 to 1995.^9^ The daily intake of folic acid during the periconceptional period was found to reduce a woman’s risk of having a foetus or infant with an NTD. The effect of this fortification was greatest in the high-prevalence rural regions. In 2003, SA legislated a programme of folic acid fortification of staple foods. An ecological study in the country, from 2002 to 2005, confirmed a significant decline in the incidence of NTDs post fortification, together with a reduction in the related financial and health burden.^10^

There are no current studies on the incidence or prevalence of NTDs in SA. Ncayiyana conducted a study from 1980 to 1984 in a rural Transkei district that showed an incidence of 6.1 NTDs/1000 births.^11^ A lower prevalence was found in urban regions such as Cape Town, namely 1.3/1000 births.^12^ According to Sayed et al.,^10^ the prevalence between January 2003 and June 2004 (pre-fortification) was 1.41/1000 births and between October 2004 and June 2005 (post-fortification) was 0.98/1000 births. This is comparable to the 1.67/1000 prevalence in low- and middle-income countries reported between 2000 and 2013.^2^

Since the study of Sayed et al.,^10^ there have been many changes in SA’s healthcare. Clinical geneticists ‘have the impression’ that there has been a resurgence of NTDs in recent years: Christianson AL, personal communication, Johannesburg, SA, May 2016. The Sayed study^10^ took place in an environment of high HIV infection rates. The slow rollout of antiretroviral treatment (ART) in the region is unlikely to have influenced the study findings. Antiretroviral treatment protocols have changed numerous times in the past 11 years. If an increase in NTDs is demonstrated, a causal relationship will need to be investigated.

The SA-National ART programme was launched in April 2004. Adult regimens comprised of two nucleoside/tide reverse transcriptase inhibitors (NRTIs) and one non-nucleoside reverse transcriptase inhibitor (NNRTI): initially stavudine and lamivudine (NRTIs) and efavirenz/nevirapine (NNRTIs).^13^ In 2010, first-line ART was changed to tenofovir and lamivudine/emtricitabine with nevirapine still preferred in women of child-bearing age.^14^ By 2013, the guideline was changed to tenofovir, emtricitabine/lamivudine and efavirenz for all adults, irrespective of gender or pregnancy.^15^ This is generally given as a fixed-dose combination tablet as recommended in the 2015 National Guidelines.^16^

An analysis in 2013 found no evidence of an increased risk of central nervous system congenital anomalies associated with first-trimester exposure to efavirenz in low- and middle-income countries.^17^ The incidence of NTDs was low and similar to that of the general population in this systematic review and meta-analysis.

Since 7 October 2003, SA legislation namely, the Foodstuffs, Cosmetics and Disinfectants Act Number 54, has required any person who manufactures, imports or sells bread, wheat-flour and maize-meal, to fortify it with vitamins and minerals.^18^ The included vitamins are vitamin A, thiamine (vitamin B1), riboflavin (vitamin B2), niacin (vitamin B3), folic acid (vitamin B9) and pyridoxine (vitamin B6). The included minerals are iron and zinc. The regulations also stipulate that a miller should keep monthly records and store the fortification mixture under hygienic conditions. Contravention of these conditions may result in a fine.^19^ Environmental Health Practitioners were trained and mobilised to carry out routine inspections of mills to ensure compliance with food fortification regulations. The success of this fortification programme depends on a multitude of factors as outlined in ‘A reflection of the South African Maize Meal and Wheat Flour Fortification Program (2004–2007)’.^19^

### Aim and objectives

This study aimed to determine if there was an increase in the incidence of NTDs in live and stillbirths in the Free State province (FS) from 2012 to 2016 as reflected by Universitas Hospital’s (UH) records.

Specific objectives:

- The incidence of NTDs found in this study was compared with the incidence of NTDs found in the FS in 2005 by Sayed et al.^10^
- Data were collected to identify recurrent factors that could be used in further research studies as possible risk factors.
- To determine the incidence of cranial versus spinal NTDs.

## Methods

This was a retrospective cross-sectional study set at UH, Bloemfontein, which is the referral hospital in the FS for the treatment of all NTDs as well as antenatal diagnoses of congenital defects. All babies with NTDs (including stillbirths, late terminations and live births) from pregnancies reaching viability born in UH or referred to UH from 2012 to 2016 were included in this study. At UH, viability is defined as later than 28 weeks’ pregnancy duration or more than 800 g birth weight.

The Department of Health Information Systems (DHIS) defines a delivery later than 28 weeks as a birth, and not a miscarriage. Early miscarriages or termination of pregnancy before 28 weeks’ gestation were excluded to enable comparison with the Sayed et al. study.^10^ Patients referred from outside the FS were also excluded.

### Measurement

Neural tube defect cases were identified and data were collected retrospectively from the following sources:

- Surveillance of Birth Defects reporting forms – UH.
- MEDITECH records: the Electronic system for patient data capturing at UH.
- Hospital records from the Neonatal unit and Obstetric unit, including admission registers as well as the records-archive of UH.
- Number of births in the FS for 2012 to 2016 – DHIS.
- The birth data for UH and Pelonomi Hospital (PH) were collected from the specific maternity wards and calculated separately to that from the rest of the FS.

Once the NTD cases were identified, data from their summaries on MEDITECH as well as hospital files were collected and entered into a data collection sheet. Each case was given a unique research number for the information of the mother and the baby to maintain the confidentiality of the patients. The data collection sheets were then entered into an Excel^®^ spreadsheet.

The first three cases were used for the pilot study to test the data collection sheet. The data-sheet was adapted to include the gender and the weight of the baby. These three cases were included in the study.

### Analysis of data

The University analysed the data. The results were summarised as frequencies, percentages (categorical variables) and medians (numerical variables due to skew distributions). Ninety five per cent confidence intervals (95% CI) and appropriate hypothesis testing were used for comparison with other studies.

### Ethical consideration

Approval to undertake this study was obtained from the Health Sciences Research Ethics Committee of the University of the Free State (HSREC 53/2016) and permission to conduct this study was also obtained from the Free State province Department of Health. The information of each patient was managed with strict confidentiality. The hospital numbers of each patient and mother and the date of birth were transcribed onto a separate list and a specific research number was allocated for each mother and baby (e.g. M1, B1). On the data-sheet and the Excel spread-sheet, only these research numbers were used to ensure confidentiality. These data-sheets were kept in a locked cabinet at the Department of Paediatrics.

## Results

**FIGURE 1:**
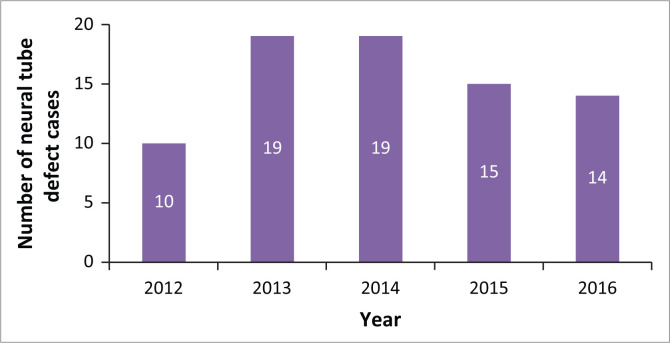
Number of neural tube defect (NTD) cases per year at Universitas Hospital.

In total, 77 cases of NTDs were captured from 2012 to 2016 at UH. The number of cases per year indicated that most were born in 2013 and 2014, with 10 in 2012, 19 in 2013, 19 in 2014, 15 in 2015 and 14 in 2016. See Figure 1.

**FIGURE 2:**
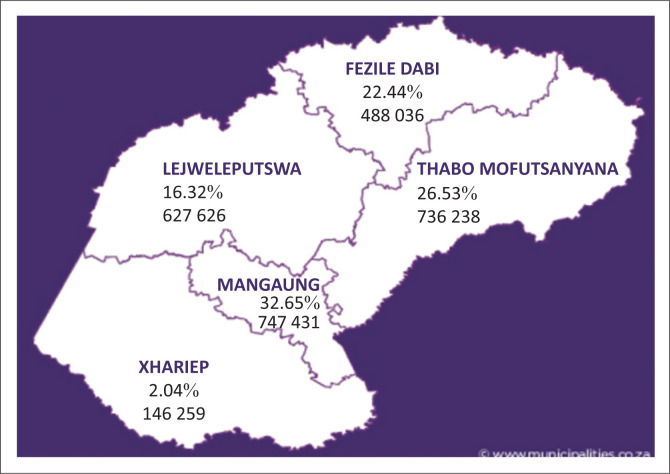
Referrals from outside of Universitas Hospital (%) by municipal district^20^ and population in 2011.^20^

Twenty-six (26; 33.8%) of the cases were born in UH, and 51 in other hospitals. The highest number of referrals was from the Mangaung area (32.7%). This is displayed in Figure 2.

The incidence of NTDs was calculated using the number of births in the FS per year as well as the number of NTDs per year, as shown in Table 1. Thus, over the 5 years, the incidence of NTDs was 0.34/1000 births.

**TABLE 1:** Birth statistics and incidence of neural tube defects in the Free State province from 2012 to 2016.

Year	Number of births	Number of NTDs	Incidence per 1000 births
2012	43 787	10	0.23
2013	44 464	19	0.43
2014	48 606	19	0.39
2015	45 982	15	0.33
2016	40 549	14	0.35

**Total**	**223 388**	**77**	**0.34**

NTD, neural tube defect.

Data for UH and PH were calculated separately from data of the rest of the FS as both had been sentinel sites for the Sayed et al. study.^10^ There were 27 222 births from 2012 to 2016 and 33 cases born at these hospitals. The incidence for NTDs was 1.21/1000 births for babies born in PH and UH, which represents the metropolitan areas of the FS. The relative risk of an NTD from 2012 to 2016 in comparison to the Sayed et al. study is 1.18 (95% CI 0.52–2.68, *p* = 0.69). There is no significant increase.

The demographics of the NTD cases revealed a male: female ratio of 1:1 with the gender known in 68 (88.3%).

Fifteen of the cases were firstborn (15; 22.4%), 20 were second-born (29.9%) and 15 third-born (22.4%). The birth order was known in 67 (87.0%).

Sixty-two (62; 80.5%) of the NTD cases were live births and 15 (19.5%) were late terminations or stillbirths.

**FIGURE 3:**
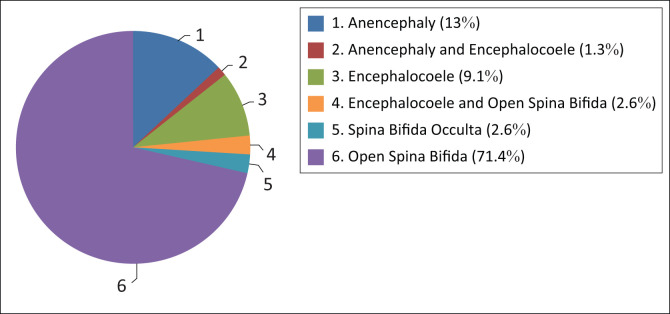
Distribution of types of neural tube defects (NTDs).

All the different types of NTDs were represented in these cases. Open spina bifida (meningocoele or myelomeningocoele) was the most common at 71.4%, 2.6% had spina bifida occulta, 13% had anencephaly and 9.1% had an encephalocoele. Some babies were reported to have more than one NTD as identified by the examining doctor: 1.3% had both anencephaly and an encephalocoele, and 2.6% had both an encephalocoele and open spina bifida. See Figure 3.

**FIGURE 4:**
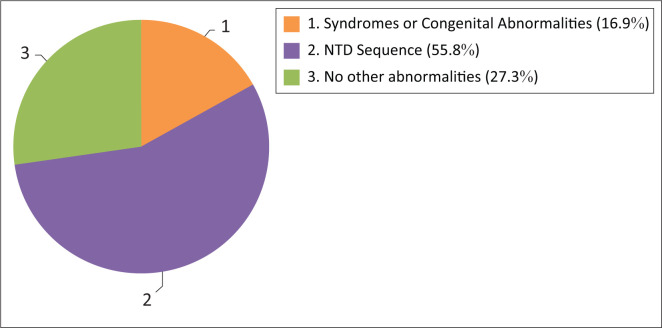
Associated congenital abnormalities.

Other congenital defects were identified in addition to the NTD in many cases. These were classified as part of the NTD sequence (found in 55.8% of cases) or part of other syndromes and associations (found in 16.9% of cases). Open spina bifida is often associated with other central nervous system anomalies, such as Chiari Type 2 malformation, hydrocephalus and club feet. These are considered as a sequence. Isolated NTDs occurred in 27.3% of cases. See Figure 4.

**FIGURE 5:**
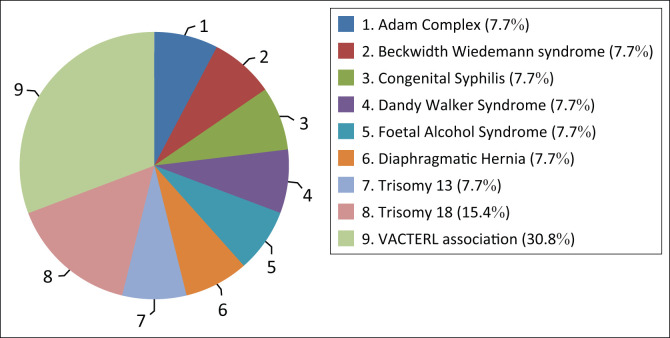
Specific syndromes or patterns of congenital abnormalities.

The other specific syndromes or patterns of congenital abnormalities most frequently associated with NTDs were VACTERL association (30.8%) and Trisomy 18 (15.4%). See Figure 5.

The data collected from the maternal files were analysed to identify risk factors that might play a role in the development of NTDs. The maternal age showed a skewed distribution with a median of 28.5 years. The maximum age was 43 years and the minimum was 16 years. The maternal age was known in 76 cases (98.7%).

Teratogen exposure was known for 63 mothers (81.8%) and 15 (23.8%) were exposed to teratogens. Three mothers were on anti-epileptic treatment, all used valproate. The epilepsy history of 67 (87.0%) mothers was known. Twelve mothers (15.6%) were exposed to other medications or toxins during their pregnancy including alcohol (*n* = 1), smoking (*n* = 1), antihypertensive treatment (*n* = 6), penicillin (*n* = 3) and traditional medicine (*n* = 1). One mother was diagnosed with diabetes mellitus during pregnancy.

**FIGURE 6:**
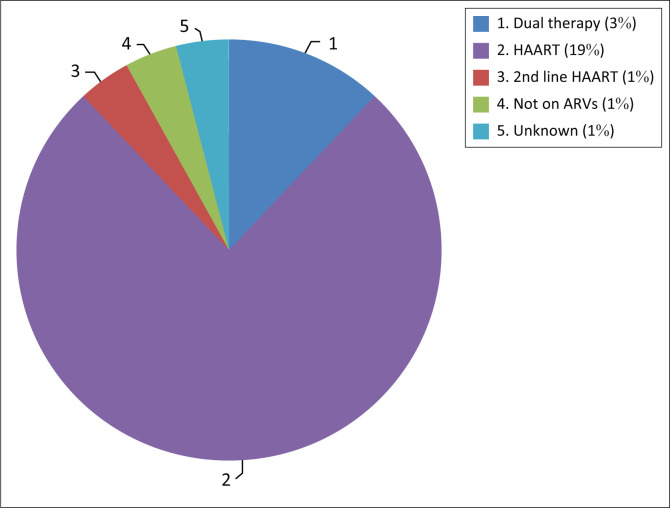
Antiretroviral (ARV) treatment of mothers who are HIV positive.

The HIV data were captured for most mothers (94.8%). The prevalence of HIV-positive mothers in this study was 34.3% (25/73). The 95% CI for the prevalence of HIV in this study was 24.4% – 45.7%, which encapsulates both the FS and national prevalence. There is, therefore, no statistically significant difference in the HIV status of mothers who delivered babies with NTDs and the general population. Of the mothers who were living with HIV, most were taking antiretroviral drugs (ARVs) (*n* = 23), one mother was not on ARVs during pregnancy and the treatment status of one mother was unknown. See Figure 6.

Family history, including previous pregnancy outcomes, was available for 34 cases (44.2%). One mother had a previous pregnancy-related NTD. Neural tube defects in the extended family were absent in the 17 cases where the data were recorded.

One mother of seven with known data had used folic acid during her pregnancy. It was unknown whether any of the mothers used folic acid during the peri-conception period.

## Discussion

This study determined the incidence of NTDs in the FS from 2012 to 2016 and looked at the maternal and foetal profiles of the babies with these defects. According to the authors’ knowledge, this is the first study to determine the incidence of NTDs in the FS. Other available data in SA are for rural Transkei (6.1 NTDs/1000 births in 1980–1984)^11^ and Cape Town (1.3/1000 in 1994)^12^. Sayed et al.^10^ used only the number of births at UH and PH, and the number of babies with an NTD born in these sentinel sites to obtain an incidence of 1.03/1000 for the FS and an incidence of 0.98 for SA using sentinel sites in three other provinces.

Compared to the above incidences, the FS incidence of 0.34/1000 found in this study is low. As only the cases referred to UH were captured, it can be postulated that this incidence is a false representation of the actual situation. Babies may die before referral or not been referred to as in the case of anencephaly, or the cases with spina bifida occulta may have been missed. An attempt was made to supplement the data with data from the DHIS where the causes of death are captured. This was unsuccessful. The entries were either not made or wrongly entered as searches indicated one death per year due to NTDs.

Comparing the incidence for UH and PH to the Sayed et al.^10^ data indicated an increase in the incidence of NTDs from 1.03/1000 in 2004/2005 (shortly after food fortification with folic acid was introduced) to 1.21/1000 in 2012 to 2016. This tends to support the impression that there is a resurgence in the number of NTD cases in the FS, but this was not statistically significant.

The referrals from outside UH were classified according to the municipal healthcare districts where the baby was born. Most referrals were from the Mangaung area as expected from the population distribution of the FS.^20^ See Figure 2.

The demographics of the NTD cases in this study contradicted most of the other studies done in SA. Both the study performed in Cape Town over 20 years ago^12^ and a study performed in Gauteng^21^ that looked at the profile of NTD cases showed that it was more prevalent in females than males, whereas this study had an equal female to male ratio. The birth order also differed from the study performed in Gauteng^21^ where the firstborn and lastborn infants were more at risk of NTDs, whereas it was the second-born infants who were mostly affected in this study. A possible explanation for the contradictions would be our small sample size.

The distribution of the type of NTD is in keeping with other studies.^2,10,11,22^ A study performed in Tunisia^22^ also showed that spina bifida was the most common defect, followed by anencephaly. The difference between the two was much smaller, however, (38.9% for spina bifida, 22.8% for anencephaly) and this can be accounted for by the fact that most anencephaly patients are probably not referred from the peripheral hospitals due to the poor prognosis.

When interpreting the maternal data, it is important to note that the data available for the mothers were poor. They were collected from the neonatal and obstetric summaries found in the electronic data-keeping system as well as the UH admission books, which were often incomplete. Most of the summaries regularly did not include the family history of NTDs or whether the mother used supplements before or during pregnancy. The data for the mother’s medication history and other chronic diseases (e.g. diabetes mellitus) that could be risk factors were also poorly represented. It is, thus, not possible to draw definitive conclusions. If the data were not recorded in the summaries, these were probably not considered whilst the patient was admitted to the hospital and the mother was, thus, also not properly counselled regarding recurrence and prevention in future pregnancies. This should be addressed by the different departments to optimise patient care.

The HIV data were captured for most mothers. HIV status was known for 94.8%. The prevalence of HIV-positive mothers in this study was 34.3%. This is higher than the prevalence in pregnant females in the FS from 2009 to 2013 which ranged between 29.8% (2013) and 32.5% (2011) according to ‘The 2013 National Antenatal Sentinel HIV Prevalence Survey South Africa’.^23^ The overall HIV prevalence in pregnant females in SA in 2013 was 29.7%, which is also lower than the prevalence found in this study. These differences were, however, not statistically significant.

The type of ARVs and the duration of ARV use were also poorly captured in the data sources. It is, therefore, not possible to say whether a specific type of ARV or a certain treatment regimen contributed to a higher rate of NTDs.

On 21 May 2018, after the completion of this study, the FDA issued a warning that women treated with dolutegravir in the first trimester of pregnancy are at higher risk for NTDs.^24^ This report also indicated that the National Institutes of Health has launched an international study to compare the safety and efficacy of three ARV treatments for pregnant women with HIV.

A recent study highlights the need for proper data surveillance and recording of congenital disorders to accurately demonstrate the contribution thereof to the burden of disease on our health care system.^25^ The Congenital Disorder Surveillance was already implemented in SA in 1980 with several changes to the system in 2001 namely, the Birth Defect Notification Tool of the National Department of Health, and a coding classification added in 2006. It was found in the study performed by Lebese et al.^25^ that the implementation of these systems was poor. When compared to expected congenital disorder notifications, there was an underreporting of more than 99%. Kwa-Zulu Natal recorded the highest number of congenital disorders per year (total of 7219 over 9 years) whilst the FS only recorded 744 cases during the same period. This highlights the need for training of healthcare providers as well as coordinators to report congenital defects so that relevant health policies can be developed. The poor data availability in this study highlighted the same limitations and issues.

## Conclusion

There is a clinical impression that the incidence of NTDs is increasing. The data obtained in this study appear to support this impression, but no significant statistical difference could be proven.

A major finding of this study was, however, the poor data capturing and recording in the FS, which will impact the planning and funding for the healthcare of congenital defects in the FS and SA as a whole.

The incidence for NTDs in the FS was found to be 0.34/1000 births, which is low compared to other South African and international data.^1,2,10,11,12,22^ The discrepancy between the incidence of NTDs in the FS and the metropolitan hospitals serves as a marker of the poor data recording.

The incidence for UH and PH for 2012 to 2016 (1.21/1000 births) is comparable to the incidence found in the Sayed et al. study.^10^ No specific correlation could be drawn between known risk factors and the NTD cases.

A prospective study in this field, with a larger study population, will be required to confirm or refute the clinical impression that there is an increase in the incidence of NTDs in the FS and to identify possible explanations, if so.
